# Supplementary figures and images for: Effects of Fluctuating Thermal Regimes on Life History Parameters and Body Size of Ophraella communa
Source: Insects. 2022 Sep 9;13(9):821. doi: 10.3390/insects13090821 (PMC9504774; doi:10.3390/insects13090821)

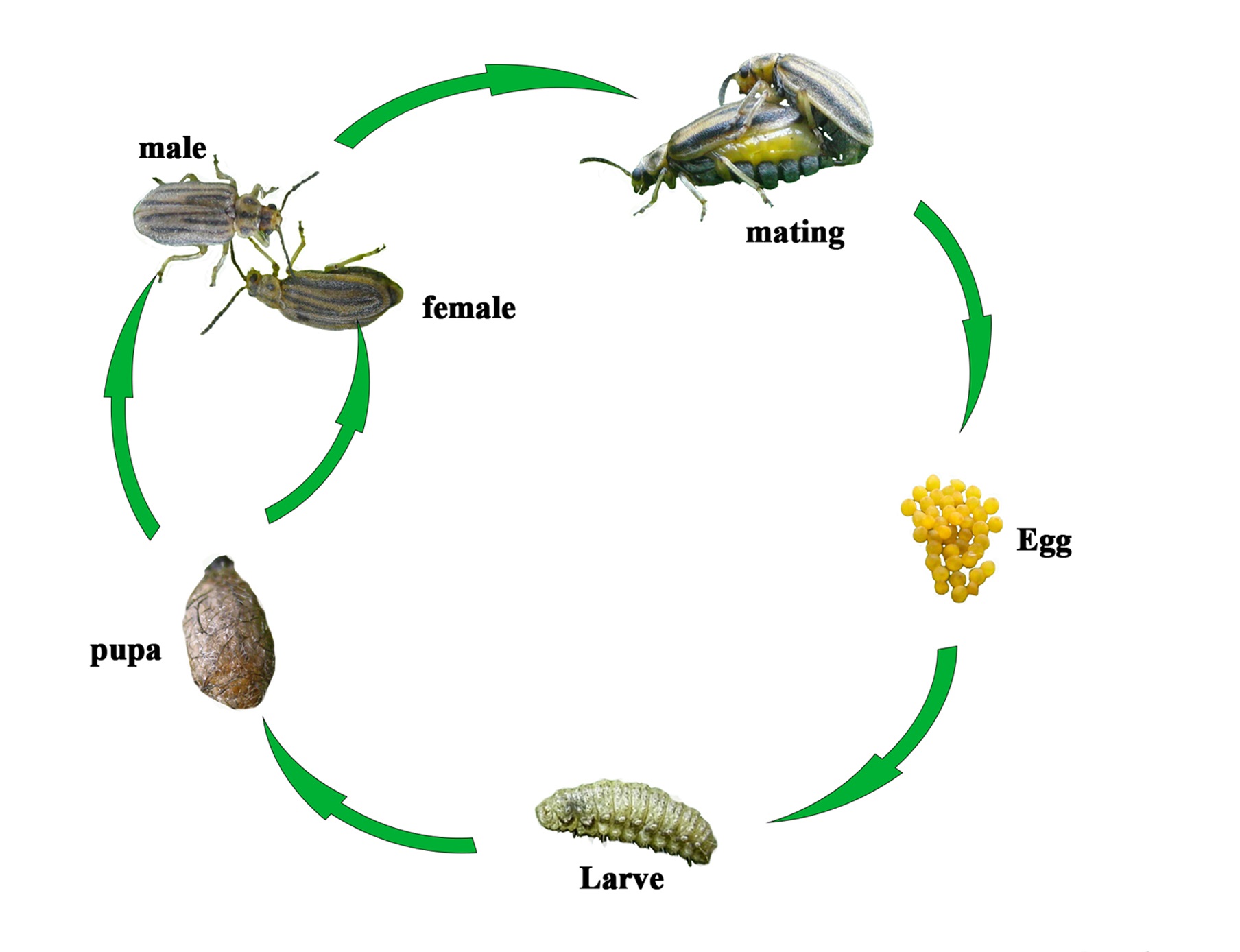

Supplement: Supplementary file 1 [file insects-13-00821-s001.zip › insects-1898371-supplementary.jpg]
